# Liquid perfluorochemical-supported hybrid cell culture system for proliferation of chondrocytes on fibrous polylactide scaffolds

**DOI:** 10.1007/s00449-014-1143-3

**Published:** 2014-02-16

**Authors:** Maciej Pilarek, Iwona Grabowska, Ilona Senderek, Michał Wojasiński, Justyna Janicka, Katarzyna Janczyk-Ilach, Tomasz Ciach

**Affiliations:** 1 Biotechnology and Bioprocess Engineering Division, Faculty of Chemical and Process Engineering, Warsaw University of Technology, Waryńskiego 1, 00-645 Warsaw, Poland; 2 Department of Cytology, Institute of Zoology, Faculty of Biology, University of Warsaw, Miecznikowa 1, 02-096 Warsaw, Poland

**Keywords:** Liquid/liquid culture system, Perfluorochemical (fluorocarbon), Polylactide (PLA), Fibrous scaffold, Electrospinning, Chondrocytes

## Abstract

CP5 bovine chondrocytes were cultured on biodegradable electrospun fibrous polylactide (PLA) scaffolds placed on a flexible interface formed between two immiscible liquid phases: (1) hydrophobic perfluorochemical (PFC) and (2) aqueous culture medium, as a new way of cartilage implant development. Robust and intensive growth of CP5 cells was achieved in our hybrid liquid–solid–liquid culture system consisting of the fibrous PLA scaffolds in contrast to limited growth of the CP5 cells in traditional culture system with PLA scaffold placed on solid surface. The multicellular aggregates of CP5 cells covered the surface of PLA scaffolds and the chondrocytes migrated through and overgrew internal fibers of the scaffolds. Our hybrid culture system simultaneously allows the adhesion of adherent CP5 cells to fibers of PLA scaffolds as well as, due to use of phase of PFC, enhances the mass transfer in the case of supplying/removing of respiratory gases, i.e., O_2_ and CO_2_. Our flexible (independent of vessel shape) system is simple, ready-to-use and may utilize a variety of polymer-based scaffolds traditionally proposed for implant development.

## Introduction

As it is commonly known, ensuring a sufficient level of mass transfer is a crucial issue in tissue engineering; otherwise, necrosis of cells within an implant could occur. If nutrient and oxygen supply, as well as wastes and carbon dioxide removal, cannot be maintained constantly, the development of an implant in each of culture system will be limited or impossible [1–3].

A cartilage engineered in vitro is usually formed by chondrocytes isolated from articular cartilage, which grows in high-density matrix composed of glycosaminoglycans and collagen. In addition to biochemical and biomechanical properties of cultured cartilage constructs, from the bioprocess engineering point of view, such specific cultures with a high matrix-to-cell-volume ratio usually exhibit mass transfer limitation. Enhancing the oxygen access to chondrocytes, as well as CO_2_ elimination, may provide appropriate conditions for tissue-engineered cartilage constructs [2, 4, 5]. There is an ongoing debate about the oxygen supply optimal to cultivate the necessary chondrocytes. However, some of the recent studies on engineered cartilage implants have reported that for isolated chondrocytes the oxygen consumption rate increases with culture time [6, 7]. Therefore, the increase in oxygen consumption rate cannot be in conflict with a culture of chondrocytes proceeding in vitro under reduced oxygen atmosphere. Even if such culture conditions are in accordance with the typical physiological range of oxygen tensions observed in vivo for natural articular cartilage [8, 9]. Additionally, implants containing living mammalian cells are usually susceptible to hydrodynamic shear stress. Therefore, in vitro systems cannot be aerated traditionally (e.g., by bubble or air-lift aeration) which is commonly applied to cultures of suspended cells. Thus, the application of liquid carriers of respiratory gases might be an alternative to conventional aeration systems [3, 10] and scavengers of CO_2_ [11, 12], as a way to increase cell growth intensity.

Synthetic liquid perfluorochemicals (PFCs), which dissolve gases according to Henry’s Law (*c*_gas_ = *H·p*_gas_), can be used as carriers of respiratory gases (i.e., O_2_ and CO_2_). Moreover, the gas transfer rate into PFCs increases linearly with the partial pressure of a component in the gaseous phase [13–20] in contrast to the sigmoid dissociation curve characteristic for biological O_2_/CO_2_ carriers. Importantly, liquid PFCs are immiscible with aqueous media and they create a separate liquid layer below the medium, at the bottom of a culture dish. The lack of chemical bonds between O_2_, CO_2_ and PFC molecules allows the efficient release of gases into the aqueous phase. The lack of toxicity and negative side effects of liquid PFCs on living cells has been confirmed by in vitro experiments and also in clinical investigations [21–27].

An innovative bioengineering application of liquid PFCs is the liquid/liquid (PFC/medium) culture system for in vitro cultures of 3-D aggregated animal cells. Such aggregates of various mammalian cells were previously cultured directly on the liquid/liquid interface created between immiscible hydrophobic PFC and aqueous culture medium [23, 27] as well as on the monolayer of collagen molecules located between the two liquid phases [24]. The basic aim of our study was to show the feasibility of the liquid/liquid culture system to inoculation and further growth of chondrocytes on biodegradable, fibrous polylactide (PLA) scaffolds which has been placed on interfacial area of the PFC/medium system as the new way of a cartilage implant development. The Food and Drug Administration (FDA) has approved PLA for a variety of biomedical applications. The electrospun PLA scaffolds exhibit high area-to-volume ratio and thus provided more surface area for cell attachment as compared to 3-D scaffolds made using other techniques [28–30]. PLA-based scaffold was previously investigated as a synthetic polymeric biomaterial for chondrocyte implant development, whose properties, such as mechanical strength, degradation rate and dimensions can all be easily controlled [31–34]. Especially, the fibrous PLA scaffolds produced by electrospinning process are considered as interesting biomaterial in bioengineering of cartilage tissue [32, 33, 35].

To our knowledge, this is the first report of the application of a culture system containing perfluorinated liquid carrier of respiratory gases in the future cartilage implant development. This is probably also the first report on the use of liquid PFCs in tissue engineering in general.

## Materials and methods

### CP5 cells and culture medium

The CP5 cell line, the anchorage-dependent, bovine (*Bos taurus*, Holstein-Fresian) articular cartilage progenitor cells [34] was used in this work. The CP5 cells have been purchased from European Collection of Cell Cultures (ECACC)/Health Protection Agency Culture Collection (Salisbury, United Kingdom). The CP5 chondrocytes were maintained in Dulbecco’s modified Eagle medium:nutrient mixture F-12 (DMEM:F12) supplemented with GlutaMAX^®^, 10 % fetal calf serum (FCS), 50 μL mL^−1^ ascorbate, and antibiotics (0.05 μL mL^−1^ penicillin, 0.05 μL mL^−1^ streptomycin) at 37 °C, and 5 % of CO_2_ according to procedure obtained from supplier of cell line [34]. The culture medium, FCS and all chemicals were obtained from Invitrogen Co. (USA) and were of animal cell culture quality.

### Liquid PFC

Perfluorodecalin (PFD; C_10_F_18_; ABCR GmbH & Co. KG, Karlsruhe, Germany) was used as a liquid carrier of respiratory gases, i.e., O_2_ and CO_2_. PFD was sterilized by autoclaving, cooled to 37 °C, and finally filtered using membrane filters (0.2 μm; Sartorius, Germany) to remove any solid contaminations. Then PFD was saturated by compressed atmospheric air in aseptic conditions to prevent any microbial contamination [16, 21, 27]. PFD created separate phase on the bottom of 24-well plates (24-WP) after pipetting components (PFD and DMEM:F12) of the liquid/liquid system as it cannot mix with aqueous systems. Thus, the volume of PFD added to the culture system does not change the concentration of DMEM:F12 ingredients [26, 27, 36].

The used PFD did not disperse and remained at the bottom of wells during all experiments. After experiments PFD was collected, recovered by filtration to remove solid remnants, then washed with ethanol and deionized water, and reused after sterilization and gas saturation procedure [25–27]. The culture system which was not supplemented with PFD, i.e., cells culture in standard medium using solid bottom surface of 24-WP, was used as the reference.

### Fibrous PLA-based scaffold

The fibrous PLA-based scaffolds have been produced by electro-hydro-dynamic atomization (EHDA) process, also known as electrospinning [37, 38]. The granulated PLA with molecular weight of 20–30 kDa and density of 1.25 g mL^−1^ was obtained from Polysciences, Inc., (USA). Prior to electrospinning, the PLA was dissolved in dichloromethane:*N*,*N*-dimethylformamide (9:1, v/v) at 7 %. To obtain electrospun mats, the polymer solution was placed in a 20-mL syringe equipped with a stainless steel needle (0.5 mm inner diameter) and extruded at 0.03 mL min^−1^. The tip of needle connected to the electrode was located 15 cm away from the collector counter-electrode. The counter-electrode was a steel plate (70 × 100 mm) covered with aluminium foil and was grounded. A high-voltage DC generator was used to supply 17 kV. The electrospun PLA mats made of 1.0–2.0 μm fibers were dried at room temperature overnight to vaporize the remaining solvent. The fibrous PLA scaffolds (16 mm diameter) were cut from dried electrospun mats. Next, PLA scaffolds were washed and sterilized by immersion in 70 % ethanol and dried in aseptic air for 1 h at room temperature.

### Experimental procedure

The CP5 cells were cultured in 24-WP (Becton-Dickinson, USA) in DMEM:F12. Three independent culture systems has been inoculated: (1) the solid surface of well’s bottom made of polystyrene and (2) fibrous PLA scaffold placed directly on the bottom of the well (i.e. control cultures), (3) fibrous PLA scaffold placed on the flexible interfacial area between PFD and DMEM:F12 (i.e. hybrid liquid–solid–liquid culture system). In both cases of control cultures the chondrocytes were cultured in 1 mL of DMEM:F12. In the case of culture system concerning liquid/liquid interface, 1 mL of PFD and 1 mL of DMEM:F12 were poured into wells to obtain flexible liquid/liquid interface and then PLA scaffold has been placed between the two liquid phases. Such procedure prevents air-bubbles entrapment in the pores of PLA scaffold.

Inoculum of CP5 cell line was prepared from standard nearly, i.e. 75–80 %, confluent cultures, passaged every 4–5 days. Briefly, cells were washed with PBS (Invitrogen Co.; USA), incubated in 0.05 % trypsin (Invitrogen Co.; USA) for ca. 3 min at 37 °C. Next, the number of cells was estimated using Malassez hemocytometer (Brand, Germany), then cells were suspended in DMEM:F12 and pipetted into the 24-WPs to obtain initial density of 2 × 10^5^ cells mL^−1^.

### Imaging methods

The fibrous PLA-based scaffolds and implants were analyzed with the Phenom scanning electron microscope (SEM) supported with image software (FEI, USA). All SEM samples were incubated in 0.5 % OsO_4_ for 1 h at 4 °C, then dehydrated/desiccated with anhydrous ethanol, next automatically critical point dried (Leica EM CPD300, Germany), and finally coated with 15-nm layer of gold (K550 Emitech, USA) prior to SEM analysis.

Live/Dead Cell Staining Kit (Sigma Aldrich, USA) was utilized for simultaneous fluorescence staining and visualization (Nikon Eclipse *Ti* microscope) of viable and dead cells.

The implants were also analyzed with the Zeiss Axiovert 100 M confocal laser scanning microscope (CLSM) supported with LSM 510 META software (Carl Zeiss Jena GmbH, Germany). CP5 cells were stained (500 ng mL^−1^) with phalloidin—tetramethylrhodamine B isothiocyanate (phalloidin-TRITC; Sigma, USA) to identify filamentous actin and with DRAQ5™ (Biostatus, UK) intercalating anthraquinone to visualize chromatin. Each imaging analysis was performed at least three times using material collected during three independent experiments.

## Results

### Hybrid liquid–solid–liquid culture system

**Fig. 1:**
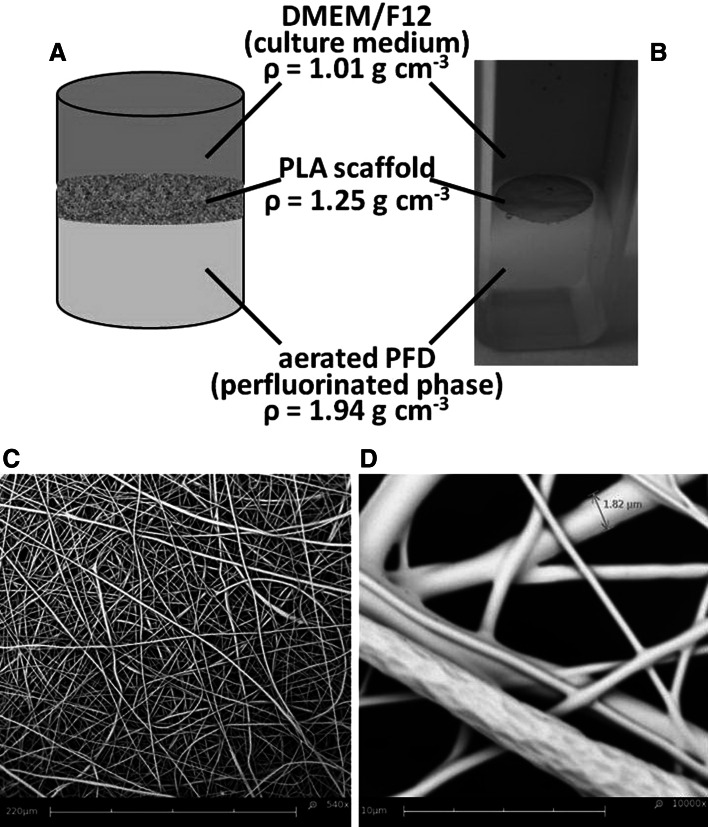
The hybrid liquid–solid–liquid culture system containing liquid phase of air-saturated PFD, solid phase of fibrous PLA scaffold and liquid phase of DMEM:F12 medium: the outline (**a**), the example of hybrid culture system (**b**), SEM microphotography of PLA scaffold (**c**; *scale bar* 220 μm) and magnified fibrous PLA scaffold with marked diameter of single electrospun PLA fiber (**d**; *scale bar* 10 μm)

An example of the hybrid liquid–solid–liquid culture system has been presented in Fig. 1. The electrospun fibrous PLA scaffold was localized at the interfacial area of two immiscible liquid phases, at the hydrophilic side of the interface. Significant differences in density of all three components (*ρ*_PFD_ = 1.94 g cm^3^, *ρ*_PLA_ = 1.25 g cm^3^, *ρ*_DMEM:F12_ = 1.01 g cm^3^) are the reason providing spontaneous, easy and self-organization of the hybrid liquid–solid–liquid system, what was in accordance with the buoyancy force affected on the used biomaterial.

### Culture of CP5 chondrocytes on fibrous PLA scaffold

**Fig. 2:**
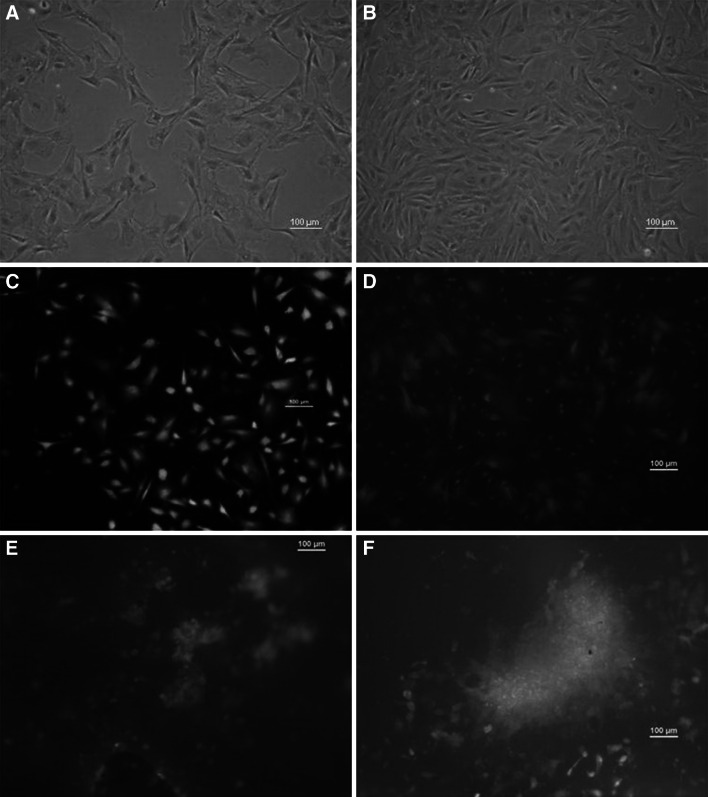
The comparison of the cells morphology of CP5 cells cultured in monolayer: 1st (**a**) and 7th (**b**) day of culture; cells cultured on the PLA scaffold placed directly on the bottom of the well (i.e. the control culture): 1st (**c**) and 7th (**d**) day of culture; cells cultured in the hybrid liquid–solid–liquid culture system: 2nd (**e**) and 7th (**f**) day of culture. **a**, **b** cells without staining; **c**, **d**, **e**, **f** live/dead fluorescence stained cells (*green* and *orange* colors for viable and dead cells, respectively). All *scale bars* 100 μm (color figure online)

CP5 cells adhered to the solid surface of 24-WP after 5–6 h (Fig. 2a). After 6–7 days cells formed a confluent monolayer (Fig. 2b).

CP5 cells cultured on PLA scaffold adhered to the PLA fibers within 8–10 h of the adaptation phase (Fig. 2c). Analysis of the Life/Dead staining performed after 7 days of culture revealed that proliferation of CP5 chondrocytes situated deeper in the fibrous PLA scaffold is rather limited and some of the cells were dead (Fig. 2d). However, definitely most of the cells located near the edge of scaffold remained flat and elongated.

After 2 days first multicellular aggregates were visible in the structure of PLA scaffold (Fig. 2e). After 7 days the expanding multicellular aggregates of proliferating CP5 cells reached about 500–700 μm diameter (Fig. 2f).

### Analysis of CP5 chondrocytes growth on fibrous scaffold in hybrid liquid–solid–liquid system

**Fig. 3:**
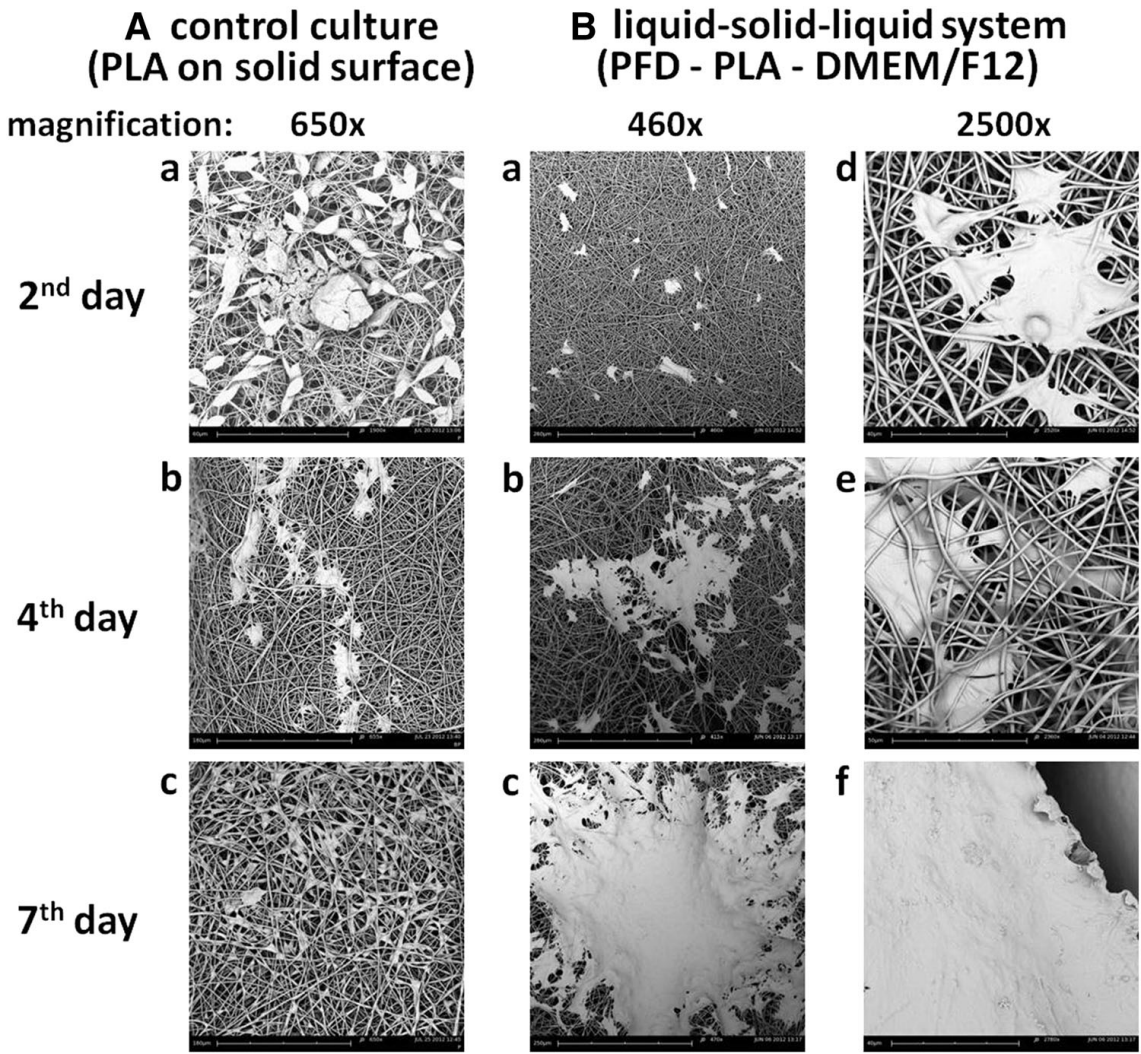
SEM micrographs documenting the morphology of CP5 cells cultured on the PLA fibrous scaffold placed directly on the solid polystyrene surface of culture plate, i.e. the control culture **A**: 2nd (a), 4th (b) and 7th (c) day of culture; and cells cultured in the PFD supported hybrid liquid–solid–liquid culture system (**B**): 2nd (a, d), 4th (b, e) and 7th (c, f) day of culture. *Scale bar* represents: **A**a **A**b **A**c 180 μm, **B**a **B**b **B**c 250 μm, **B**d **B**e **B**f 40 μm

CP5 cells were cultured under conditions supporting their proliferation. SEM analysis of the inoculated PLA scaffolds has showed that CP5 chondrocytes adhered strongly to fibers of PLA scaffolds taken from the control culture (Fig. 3A a) and from the hybrid liquid–solid–liquid culture system (Fig. 3B d, e). However, in the case of the control culture, after 7 days the growth of cells was limited and multicellular aggregates on the surface of PLA scaffold were not observed (Fig. 3A c). This is consistent with the data presented in Fig. 2d, where besides the living cells the presence of dead chondrocytes has been revealed. In the hybrid liquid–solid–liquid culture system the CP5 cells formed multicellular aggregates on the surface of fibrous PLA-based scaffold (Fig. 3B b, c). Locally, outer area of the PLA scaffold was completely covered by the expanding multicellular aggregates of proliferating CP5 cells (Fig. 3B f) which has been shown in Fig. 2f.

**Fig. 4:**
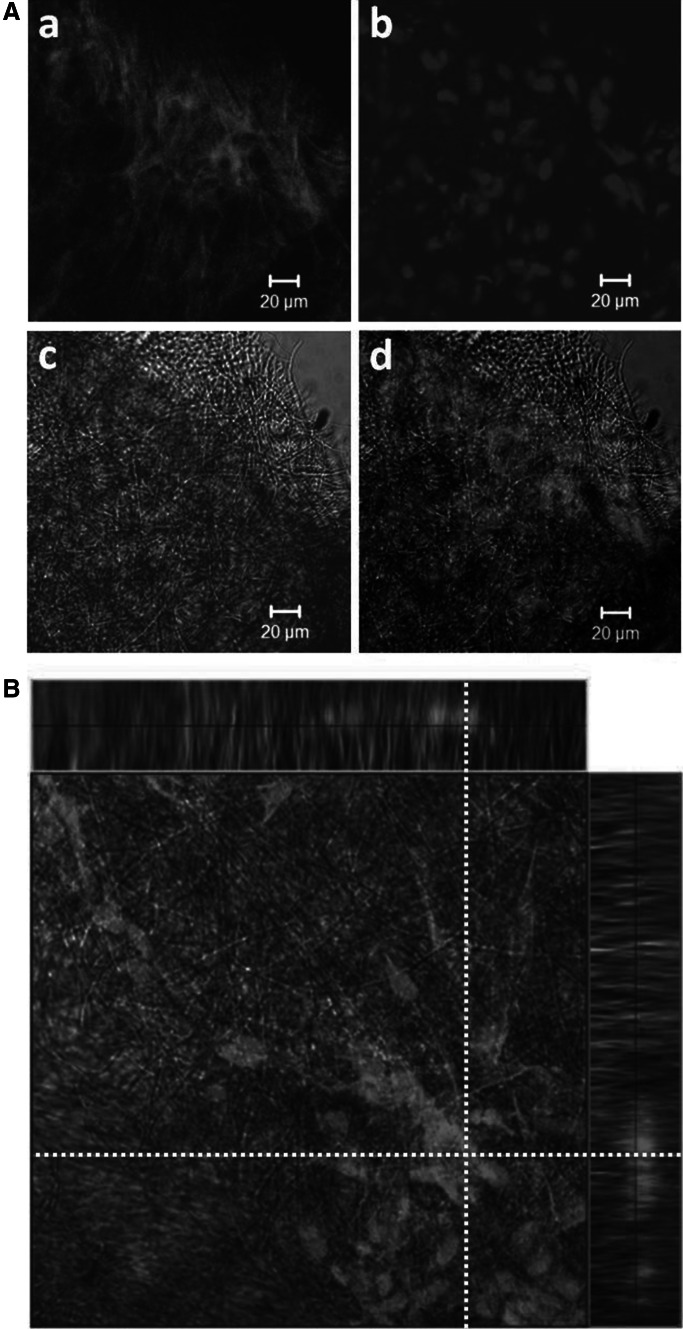
CLSM micrographs of CP5 chondrocytes growing inside the fibrous PLA scaffold taken from the liquid–solid–liquid culture system (7th day of culture): CP5 cells stained for actin molecules (**A**a) and the cell nucleus DNA (**A**b), the PLA fibers visible in transmitted light (**A**c) and image of CP5 cells on the PLA scaffold (**A**d); overview of the focal plane inside the PLA scaffold (**B**) (analyzed focal plane has been marked as *white-dotted lines*). All *scale bars* 20 μm (color figure online)

The CP5 cells progressively invaded the outer area of PLA scaffold as presented in Fig. 3B c and B f. CLSM micrographs of the PLA scaffold taken from the hybrid liquid–solid–liquid culture system have been prepared to confirm proliferation of the CP5 chondrocytes on the scaffold and to prove migration and growth of the cells also inside the scaffold. Specific fluorescent staining of the filamentous actin, as an element of cytoskeleton, has been presented in Fig. 4A a and staining of the cell nucleus DNA has been shown in Fig. 4A b. Overlapped images of actin, chromatin and the PLA fibers (Fig. 4A c) explicitly indicated that the multicellular aggregates of CP5 cells covered the PLA scaffold (Fig. 4A d). CLSM visualization of the CP5 chondrocytes (Fig. 4b) proved that the CP5 cells ingrowth proceeded from the top to the bottom of the PLA scaffold taken from the hybrid liquid–solid–liquid culture system. However, the deposition of CP5 cells was higher in the upper part of the scaffold than in the lower one.

## Discussion

In our hybrid liquid–solid–liquid culture system the PLA scaffold has been localized at the interfacial area of two immiscible liquid phases, at the hydrophilic side of the interface. Such easy self-organization of the culture system was possible according to difference in densities of its all three components (i.e. *ρ*_PFD_ = 1.94 g cm^−3^, *ρ*_PLA_ = 1.25 g cm^−3^, *ρ*_DMEM:F12_ = 1.01 g cm^−3^) and the buoyancy force. Therefore, any scaffold characterized by value of density which is simultaneously lower than density of PFD (i.e. 1.94 g cm^−3^) and higher than density of utilized culture medium (i.e. about 1.01 g cm^−3^) can be used to prepare particular hybrid liquid–solid–liquid system. The hybrid culture system presented here can be used in culture vessel of any shape (e.g., multi-well plate, dish, flask, etc.) and is also fully scalable in the case of volume and area of utilized culture vessel. What is also important from the feasibility point of view, the cell/tissue implant obtained in our perfluorochemical-supported hybrid liquid–solid–liquid culture system is ready for further use (e.g., for following chondrogenesis induction) without any need for enzymatic treatment or mechanical operations. An implant can be just simply manually raised from the interfacial area of PFD and culture medium, and transferred into a destination with maintaining of the 3-D structure of cells/aggregates.

In the hybrid liquid–solid–liquid culture system presented in current work all nutrient components required by CP5 cells to growth were supplied by the phase of culture medium, i.e. DMEM:F12. The liquid phase of air-saturated PFD enhanced mass transfer in the whole culture system and it had dual function: (1) additional reservoir of O_2_ and (2) scavenger of CO_2_ generated by cells. The PLA scaffold was directly accessible for anchorage-dependent CP5 cells to promote their adhesion after inoculation. Thus, our hybrid culture system simultaneously allows the adhesion of adherent CP5 cartilage cells to fibrous of PLA scaffold as well as phase of PFD enhances the mass transfer in the case of supplying/removing of respiratory gases, i.e. O_2_ and CO_2_.

Chondrocytes, as the CP5 cells, has been chosen to perform the experiment because they are usually in vitro cultured in high density of glycosaminoglycan and collagen matrix [2, 5], and there is a distinct need to develop the alternative techniques of mass transfer intensification within such specific implants to prevent the necrosis of the cells. Although there’s an ongoing debate about the oxygen supply optimal to cultivate chondrocytes necessary, we hypothesize that using such a PFC-supported hybrid liquid–solid–liquid culture system could have significant consequences for procedures involving transplantation of cultured human autologous chondrocytes as well as other kind of cells. As a result of this, O_2_ transfer, as well as CO_2_ transfer, which are the limiting factors in all of high-cell density cultures of mammalian cells, especially those having a constant and high oxygen demand (e.g., hepatocytes), could be excluded. The efficient solubility of range of polar gases in liquid PFCs hypothetically enables to apply our hybrid culture system for testing of cell growth in the presence of any gaseous compounds (e.g., in chondrogenesis studies, when chondrocytes prefer low oxygen concentration).

As it has been presented in the results section, intense growth of CP5 cells and cell-cluster formation inside fibrous PLA scaffolds has been achieved in the hybrid liquid–solid–liquid culture system, in contrast to limited growth of cells in the reference culture system. Positive effects of PFC application on mammalian cell growth have been reported previously by few research groups [23, 24, 27]. In our experiments PFD has been saturated with atmospheric air to prevent chondrocytes from oxygen tension out of physiological range [27]. From the bioengineering point of view, the fibrous scaffolds were located at the interfacial area, created between liquid phases of PFD and culture medium. Hydrophobic PFD could easily fill up internal cavities of part of hydrophobic fibrous PLA scaffolds which is immersed in PFD. We hypothesize that such wettability of fibrous PLA scaffold by PFC with high affinity to respiratory gases had a positive influence on mass transfer intensification (in the case of O_2_ supply and CO_2_ remove) inside biomaterial structure and finally promoted the CP5 cells proliferation and formation of cell-aggregates/clusters within the scaffold.

The results of the analyses of CP5 chondrocytes proliferation within fibrous PLA scaffold in the hybrid liquid–solid–liquid culture system presented in the current work are probably the first report of the application of such specific culture system in cartilage implant development. Because of this, data presented here cannot be compared with data on chondrocyte cultures on electrospun PLA scaffolds previously published by other researchers, e.g., [33, 35]. Data presented in the current work cannot be also referenced to results of other mammalian cell cultures which have been previously performed directly at PFD:medium interface (i.e. without solid-phase promoter of cells adhesion, as it has been studied in the current work) [23, 24, 27].

## Conclusions

The proposed PFC-supported hybrid liquid–solid–liquid culture system containing PLA fibrous scaffolds may have promising applications in any implant development, where the cells must be cultured in high density of cells or extracellular matrix, i.e. chondrocytes, in flat and 3-D structures. This flexible (independent of vessel shape) system is simple and ready-to-use and may be applied for any polymer-based scaffolds traditionally proposed for implant development. Robust and intense proliferation of CP5 bovine chondrocytes was achieved in the hybrid liquid–solid–liquid culture system containing the fibrous PLA scaffolds in contrast to limited growth of the CP5 cells in traditional culture system with PLA scaffold placed on solid surface. Other benefit of our system is its availability to be used for testing of the cell growth in the presence of any gaseous compounds, for example as a simple device in hypoxia or hyperoxia of mammalian cell/tissue implants in vitro studies.

## Abbreviations

CP5: Articular cartilage progenitor cell line
PFC: Liquid perfluorochemical (fluorocarbon)
PFD: Perfluorodecalin
PLA: Polylactide
